# Pupil response as a window into cognitive processing in amyotrophic lateral sclerosis

**DOI:** 10.1016/j.ensci.2025.100575

**Published:** 2025-07-10

**Authors:** Mohamad El Haj, Souheil Hallit, Claire Boutoleau-Bretonnière

**Affiliations:** a Nantes Université, Univ Angers, Laboratoire de Psychologie des Pays de la Loire (LPPL - EA 4638), F-44000 Nantes, France; b CHU Nantes, Clinical Gerontology Department, Bd Jacques Monod, F44093 Nantes, France; c School of Medicine and Medical Sciences, Holy Spirit University of Kaslik, P.O. Box 446, Jounieh, Lebanon; d Department of Psychology, College of Humanities, Effat University, Jeddah 21478, Saudi Arabia; e Applied Science Research Center, Applied Science Private University, Amman, Jordan; f CHU de Nantes, Centre Memoire Ressource et Recherche (CMRR), Departement de Neurologie, Nantes, France; g Inserm CIC 04, Nantes, France

**Keywords:** Amyotrophic lateral sclerosis, Cognition, Neuropsychological assessment, Pupil, Pupil dilation

## Abstract

**Purpose:**

This preliminary study aimed to investigate whether the pupil size reflects cognitive load in patients with amyotrophic lateral sclerosis (ALS).

**Methods:**

Pupil activity was monitored in three patients with ALS and a group of healthy control participants (*n* = 16) while performing three tasks: a forward span task, a backward span task, and a control task involving counting aloud. These tasks were designed to impose increasing cognitive demands, with the backward span task being the most challenging.

**Results:**

Analysis revealed no significant difference in pupil size between patients with ALS and controls for the forward or backward spans or the control condition. Both groups demonstrated a consistent pattern of increased pupil size during the backward span task compared to the forward span task, and during the forward span task compared to the control condition.

**Conclusion:**

These findings suggest that pupil dilation reflects task-related cognitive load similarly in ALS patients and healthy controls. This supports the use of pupillometry as a non-invasive and sensitive marker of cognitive processing in ALS.

## Highlights

• Pupil dilation was monitored in ALS patients and healthy controls during cognitive tasks.
• Both groups showed increased pupil size with higher cognitive load (backward > forward > control task).
• No significant difference in pupil response between ALS patients and controls was found.
• Findings suggest that pupillometry reliably reflects cognitive load in ALS.
• Supports the potential of pupil size as a non-invasive marker of cognitive processing in neurodegenerative diseases.

Can pupil dilation offer a window into cognitive processing in patients with amyotrophic lateral sclerosis (ALS)? This study aims to address this question by examining how cognitive processing manifests through measurable changes in pupil activity in ALS. ALS is a neurological condition marked by dysfunction of both upper and lower motor neurons, affecting the bulbar, cervical, thoracic, or lumbar regions [1]. Although traditionally considered a motor disorder, ALS is now recognized as a condition that involves cognitive changes, which frequently manifest early in the disease course [[2], [3], [4], [5], [6]]. Even in cases of mild cognitive impairment in ALS, deficits in verbal fluency and executive functioning are commonly reported [[7], [8], [9]]. Deficits in executive function in general can be associated with deficits in the central executive system of working memory [10]. This paper thus investigated whether cognitive demand can be indexed by pupil dilation in ALS.

## 1 Method

### 1.1 Participants

The study included three cases with ALS and 16 control participants. We recruited the patients from the Memory Clinic Center at the Neurology Department of the Hospital of Nantes. Case 1 was a 51-year-old man with an undergraduate degree. Case 2 was a 71-year-old man with a high school degree. Case 3 was a 62-year-old man with a high school degree. All participants met the Revised El Escorial criteria for ALS. Case 1 was diagnosed with ALS, with upper limb impairment, for two years. Case 3 was diagnosed with ALS and had been tetraplegic for eight years. The diagnosis was based on medical history, neurological examination, and electromyographic findings consistent with ALS. Exclusion criteria were 1) other neurocognitive disorders, such as Alzheimer's disease or frontotemporal dementia, 2) psychiatric disorders, 3) treatment with psychoactive drugs or other medications affecting mental status, and 4) severe motor impairments, such as upper extremity deficits or dysarthria, that could interfere with procedures.

Control participants were 16 men (*M*age in years = 64.56, *SD* = 5.10), recruited from the local community based on the lack of a history of neurological/psychiatric disorders. No significant differences were observed between the two populations regarding age [*t*(17) = 0.87, *p* = .40]. General cognitive functioning was screened using the Mini-Mental State Examination (MMSE), a widely used standardized tool for assessing global cognitive functioning. The MMSE includes items evaluating orientation, immediate and delayed recall, attention and calculation, language, and visuospatial skills. The maximum score is 30, with lower scores indicating greater cognitive impairment. This tool is often used in clinical and research settings to detect cognitive changes in neurodegenerative diseases. Higher scores on the MMSE were observed in control participants (*M* = 28.63, *SD* = 0.96) compared to patients (*M* = 24.67, *SD* = 1.53) [*t*(17) = 6.04, *p* < .001]. On the latter test, scores of Case 1, Case 2, and Case 3 were, respectively, 26, 23, and 25. The study was approved by the Committee for the Protection of Persons (the French national ethical board, approval number 20202-A02276–33). All participants provided written informed consent.

### 1.2 Procedures

The study consisted of three conditions: forward spans, backward spans, and a control task. The control task of counting aloud was chosen because it required verbal output of numbers similar to the span tasks but involved a lower cognitive load. The three conditions were also chosen as they involve no motor requirements. During all conditions, participants wore eye-tracking glasses while facing a blank white wall. The glasses (Pupil Lab) used an infrared pupil-tracking system, sampling at 200 Hz with a gaze position accuracy of less than 0.1°. The sequence of the three conditions was randomized across participants, with adjustments to the glasses made between each task. Testing was conducted individually in a quiet, distraction-free room. The environment was standardized with a consistent light source (60-watt fluorescent bulb), and the room was free of visual stimuli except for the blank white wall. Participants were seated 30–50 cm from the wall, and blinds were closed to maintain consistent lighting.

The forward and backward span tasks were adapted from the WAIS-R. In the forward span, participants were asked to repeat a sequence of digits in the same order as presented. This task primarily taps into attention and short-term memory. In the backward span, participants were instructed to repeat the digits in reverse order, which imposes greater demands on working memory and executive control. Each sequence increased in length until the participant failed to correctly reproduce two sequences of the same length. The span score reflects the maximum number of digits correctly recalled in the correct order (or reverse order for the backward task). For the control task, participants counted aloud starting from the number “1” for two minutes. All participants successfully completed this task.

Before recording pupil data, calibration was conducted by having participants fixate on a black cross (5 × 5 cm) printed on A4 paper and placed at the center of the wall. The cross was removed once calibration was complete. Pupil size was recorded from the dominant eye using eye-tracking glasses (Pupil Labs) equipped with an infrared camera sampling at 200 Hz and a gaze accuracy of <0.1°. The pupil size signal was continuously recorded throughout the duration of each task condition. Between each condition, participants were given a short resting interval of approximately one minute to minimize fatigue and allow for any necessary adjustments to the eye-tracking equipment. For each condition, pupil data were analyzed by calculating the mean pupil diameter across the full task duration, excluding artifacts such as blinks. Blinks were automatically detected as brief signal losses due to the absence of corneal reflection and were removed from the dataset. The resulting clean signal was used to compute condition-specific average pupil size in millimeters, providing a continuous measure of cognitive load during task execution.

### 1.3 Statistical analysis

We compared performances on spans between patients and control participants by calculating *Z*-scores for the patients' raw scores based on the mean and standard deviation of the control group. We also calculated Z scores to determine whether pupil size for each patient differed significantly from the control group. For the control group, differences in pupil size across all three conditions were analyzed using a one-way ANOVA, followed by paired *t*-tests to identify specific significant differences.

## 2 Results

### 2.1 Spans

Scores on spans are provided in Table 1. Compared to control participants, patients demonstrated similar performances (with *Z*-scores higher than the critical value of −1.645). Raw data comparisons showed lower performances on the backward than on the forward spans in the patients. Lower performances on the backward than on the forward spans were also observed in control participants, *t*(30) = 3.00, *p* = .005.

**Table 1:** Spans scores in the patients and control participants.

	Patient1	Patient2	Patient3	Controls
Spans forward	6, Z = 0.37	5, Z = −1.18	6, Z = 0.37	6.46 (1.24)
Spans backward	5, Z = 0.11	4, Z = −0.91	4, Z = −0.91	5.14 (1.25)

*Note.* For patients, we provided raw scores and calculated *Z*-scores with reference to the control group; for the control group, we provide means and, in parentheses, standard deviations; scores on spans referred to the number of digits in the correctly repeated last sequence.

### 2.2 Pupil size

Pupil size across conditions is provided in Table 2. For the control task, all patients had *Z*-scores above the critical threshold of −1.645, indicating no significant differences in pupil size compared to the control group. Likewise, no significant differences in pupil size were observed between patients and controls for the forward or backward conditions.

**Table 2:** Pupil size, in mm, in the patients and control participants during the control and span tasks.

	Patient1	Patient2	Patient3	Controls
Control task	2.31, Z = −0.19	2.21, Z = 0.05	2.30, Z = 0.17	2.17 (0.75)
Spans forward	2.94, Z = −0.08	3.09, Z = 0.07	3.16, Z = 0.13	3.02 (0.95)
Spans backward	3.81, Z = −0.11	3.70, Z = −0.19	3.92, Z = −0.03	3.96 (1.39)

*Note.* For all participants, we provide means, and, in parentheses, standard deviations, for pupil size during each condition; the *Z*-scores were calculated with reference to the control group.

Regarding differences between conditions, raw data comparisons show larger pupil size in the backward span than in the forward span, and larger in the forward span than in the control condition for each patient. Among control participants, significant differences in pupil size were observed across the three conditions, *F*(2, 45) = 8.20, *p* = .001. Post hoc tests revealed larger pupil sizes in the backward span compared to the forward span, *t*(30) = 2.23, *p* = .033, and in the forward span compared to the control condition, *t*(30) = 2.81, *p* = .009.

## 3 Discussion

We compared pupil activity in three patients with ALS and control participants across three tasks: forward and backward span tasks and a control task involving counting aloud. The analysis revealed no significant differences in pupil size between patients with ALS and control participants for the forward or backward spans or the control condition. However, both groups demonstrated increased pupil size during the backward span task compared to the forward span task, and during the forward span task compared to the control condition. These results indicate that pupillary responses to increasing cognitive demands remain preserved in ALS. While pupillometry did not differentiate ALS patients from controls in this preliminary sample, the fact that pupil size increases consistently with task difficulty in both groups suggests that pupillometry could be a useful, non-invasive tool to monitor cognitive function in ALS.

The primary finding of this study was the increase in pupil size observed during the backward span task compared to the forward span task and the control condition. This increase in pupil size may be attributed to the heightened cognitive load required for the backward span task, which exceeds that of the forward span task and the control condition. This interpretation aligns with prior research demonstrating that pupil size reflects cognitive load and that pupil dilation occurs in response to increased cognitive demands [[11], [12], [13]]. Notably, our analysis revealed no significant differences in pupil size between patients with ALS and control participants for the forward or backward spans or the control condition. This finding suggests that, while pupil size reliably reflects task-related cognitive load in both groups, the degree of pupil dilation in response to these tasks does not differ significantly between ALS patients and controls.

The putative relationship between pupil size and cognitive load in our study can be supported by the lower scores on backward than forward spans in both patients with ALS and control participants. These lower scores suggest that the backward span task imposes a greater cognitive demand than the forward span task, consistent with the observed increase in pupil size. The increased pupil size during the backward span task may reflect the heightened working memory and executive function demands necessary for reversing sequences, compared to the relatively lower demands of the forward span task.

These findings should also be interpreted in light of the clinical heterogeneity of ALS, particularly the distinction between limb-onset and bulbar-onset forms. Bulbar-onset ALS can be more frequently associated with cognitive and behavioral impairments, especially in executive and language domains, likely reflecting greater frontotemporal cortical involvement [4]. This variation may influence task-related pupil responses, as individuals with bulbar onset could show altered dilation patterns due to more pronounced cognitive deficits. We acknowledge that the small sample size in our study—largely attributable to the relative rarity of ALS and the challenges of recruiting well-characterized patients—limits the generalizability of our findings. Also, although there is a disparity in sample sizes between the ALS cohort and healthy controls, the use of *Z*-score transformations based on control data enables robust individual comparisons. This statistical approach mitigates the impact of sample size imbalance and supports the validity of our findings. It is also important to note that the patients in this study were likely in the relatively mild stages of the disease, as indicated by their preserved verbal fluency and scores on the MMSE and span tasks. The use of standardized disease severity measures such as the Revised Amyotrophic Lateral Sclerosis Functional Rating Scale could help quantify disease progression more precisely in future studies.

To summarize, our study highlights the potential of pupillometry as a non-invasive tool for assessing cognitive demand in ALS. By demonstrating that pupil dilation reliably reflects task demands, even in patients with neurodegenerative conditions, this study opens new avenues for clinical applications. Pupillometry could serve as a sensitive biomarker for tracking cognitive demand in ALS.

## CRediT authorship contribution statement

**Mohamad El Haj:** Writing – original draft, Investigation, Methodology. **Souheil Hallit:** Visualization, Validation, Writing – review & editing. **Claire Boutoleau-Bretonnière:** Validation, Project administration, Visualization, Supervision.

## Funding

None.

## Declaration of competing interest

The authors have no conflict of interest to declare.

## Data Availability

Raw data can be shared under reasonable request by email to the correspondent author.

## References

[1] Goutman S.A., Hardiman O., Al-Chalabi A., Chió A., Savelieff M.G., Kiernan M.C. (2022). Recent advances in the diagnosis and prognosis of amyotrophic lateral sclerosis. The Lancet Neurology..

[2] Bersano E., Sarnelli M.F., Solara V., Iazzolino B., Peotta L., De Marchi F. (2020). Decline of cognitive and behavioral functions in amyotrophic lateral sclerosis: a longitudinal study. Amyotrophic Lateral Sclerosis and Frontotemporal Degeneration.

[3] Beeldman E., Govaarts R., de Visser M., Klein Twennaar M., van der Kooi A.J., van den Berg L.H. (2020). Progression of cognitive and behavioural impairment in early amyotrophic lateral sclerosis. Journal of Neurology, Neurosurgery Psychiatry.

[4] Pender N., Pinto-Grau M., Hardiman O. (2020). Cognitive and behavioural impairment in amyotrophic lateral sclerosis. Curr Opin Neurol.

[5] Crockford C., Newton J., Lonergan K., Chiwera T., Booth T., Chandran S. (2018). ALS-specific cognitive and behavior changes associated with advancing disease stage in ALS. Neurology.

[6] Ringholz G.M., Appel S.H., Bradshaw M., Cooke N.A., Mosnik D.M., Schulz P.E. (2005). Prevalence and patterns of cognitive impairment in sporadic ALS. Neurology.

[7] Rusina R., Vandenberghe R., Bruffaerts R. (2021). Cognitive and behavioral manifestations in ALS: beyond motor system involvement. Diagnostics.

[8] Goldstein L.H., Abrahams S. (2013). Changes in cognition and behaviour in amyotrophic lateral sclerosis: nature of impairment and implications for assessment. The Lancet Neurology.

[9] Abrahams S., Leigh P.N., Harvey A., Vythelingum G.N., Grisé D., Goldstein L.H. (2000). Verbal fluency and executive dysfunction in amyotrophic lateral sclerosis (ALS). Neuropsychologia.

[10] Baddeley A. (1992). Working memory. Science.

[11] Hess E.H., Polt J.M. (1964). Pupil size in relation to mental activity during simple problem-solving. Science.

[12] Hess E.H., Polt J.M. (1960). Pupil size as related to interest value of visual stimuli. Science.

[13] Kahneman D., Beatty J. (1966). Pupil diameter and load on memory. Science.

